# Interictal Activity Is Associated With Slower Binocular Rivalry in Idiopathic Generalized Epilepsy

**DOI:** 10.3389/fneur.2021.720126

**Published:** 2021-11-10

**Authors:** Jiaonan Wu, Wei Ding, Xing Ye, Qiang Wei, Xinyi Lv, Qiqiang Tang, Yanghua Tian, Kai Wang, Yubao Jiang

**Affiliations:** ^1^Department of Neurology, The First Affiliated Hospital of USTC, Division of Life Sciences and Medicine, University of Science and Technology of China, Hefei, China; ^2^Department of Neurology, Anhui Provincial Hospital, Cheeloo College of Medicine, Shandong University, Jinan, China; ^3^Department of Nephrology, The First Affiliated Hospital of USTC, Division of Life Sciences and Medicine, University of Science and Technology of China, Hefei, China; ^4^Department of Neurology, Nanjing Brain Hospital, Nanjing Medical University, Nanjing, China; ^5^Department of Neurology, The First Affiliated Hospital of Anhui Medical University, Hefei, China; ^6^Key Laboratory of Cognition and Neuropsychiatric Disorders, Hefei, China

**Keywords:** binocular rivalry, idiopathic generalized epilepsy, electroencephalogram, excitatory neurotransmitter, inhibitory neurotransmitter

## Abstract

**Objective:** Perceptual alternations evoked by binocular rivalry (BR) reflect cortical dynamics strongly dependent on the excitatory–inhibitory balance, suggesting potential utility as a biomarker for epileptogenesis. Therefore, we investigated the characteristics of BR in patients with idiopathic generalized epilepsy (IGE) and potential associations with clinical variables.

**Methods:** Sixty-two healthy controls (HCs) and 94 IGE patients completed BR task. Perceptual alternation rates were compared between HC and IGE groups as well as among the HC group and IGE patients stratified according to the presence or absence of interictal activity on the ambulatory electroencephalogram (EEG), termed the abnormal ambulatory EEG group (AB-AEEG, *n* = 64) and normal ambulatory EEG group (N-AEEG, *n* = 30), respectively.

**Results:** The IGE patients demonstrated a slower rate of BR perceptual alternation than HC subjects (*t* = −4.364, *p* < 0.001). The alternation rate also differed among the HC, AB-AEEG, and N-AEEG groups (*F* = 44.962, *df* = 2, *p* < 0.001), and *post hoc* comparisons indicated a significantly slower alternation rate in the AB-AEEG group compared with the N-AEEG and HC groups (0.28 vs. 0.46, and 0.43 Hz). Stepwise linear regression revealed positive correlations between the BR alternation rate and both the ambulatory EEG status (β, 0.173; standard error, 0.022 *p* < 0.001) and Montreal Cognitive Assessment score (β, 0.013; standard error, 0.004; *p* = 0.003). Receiver operating characteristic curve analysis of the BR alternation rate distinguished AB-AEEG from N-AEEG subjects with 90.00% sensitivity and 76.90% specificity (area under the curve = 0.881; 95% confidence interval = 0.801– 0.961, cut-off = 0.319). Alternatively, Montreal Cognitive Assessment score did not accurately distinguish AB-AEEG from N-AEEG subjects and the area under the receiver operating characteristic curve combining the BR alternation rate and Montreal Cognitive Assessment score was not markedly larger than that of the BR alternation rate alone (0.894, 95% confidence interval = 0.822–0.966, *p* < 0.001). K-fold cross-validation was used to evaluate the predictive performance of BR alternation rate, MoCA score, and the combination of both, which yielded average AUC values of 0.870, 0.584 and 0.847, average sensitivity values of 89.36, 92.73, and 91.28%, and average specificity values of 62.25, 13.42, and 61.78%, respectively. The number of interictal epileptiform discharges was significantly correlated with the alternation rate in IGE patients (*r* = 0.296, *p* = 0.018). A forward stepwise linear regression model identified the number of interictal epileptiform discharges (β, 0.001; standard error, 0.001; *p* = 0.025) as an independent factor associated with BR alternation rate in these patients.

**Conclusion:** These results suggest that interictal epileptiform discharges are associated with disruptions in perceptual awareness, and that the BR may be a useful auxiliary behavioral task to diagnosis and dynamically monitor IGE patients with interictal discharge.

## Introduction

When incongruent images are presented to the two eyes, perceptual awareness spontaneously alternates every few seconds between one image and the other rather than forming a stable composite (1–3). This phenomenon, known as “binocular rivalry” (BR), is mediated by competitive interactions between populations of neurons that code for the two inputs at various levels of visual processing.

Binocular rivalry is a specific example of a more general phenomenon termed bistable perception. Since the stimulus remains constant, the spontaneous perceptual alternation characteristic of bistable perception reflects inherent dynamic operations in the brain and thus may offer a tool to distinguish normal from abnormal neural dynamics. Functional imaging techniques and electrophysiology have revealed that as the perceptual awareness of one image (precept) is suppressed, there is a concomitant reduction in response amplitude within brain regions processing that image, and that this reduction is reversed as the percept regains dominance (4, 5). Brain imaging studies also indicate that bistable perception is associated with shifting neural dynamics within both early visual areas (6) and higher centers such as frontal and parietal cortex (7–11). Transcranial magnetic stimulation studies by Carmel and colleagues suggested that activity in parietal cortex reflects the rate of bistable perception (10, 12, 13). Thus, the dynamics of these perceptual changes can be utilized to infer underlying neural dynamics (14, 15). The neural dynamics reflective of BR critically depend on the balance between excitation and inhibition (E/I balance) in the cortex (2, 16–20), suggesting that the characteristics of BR may change in pathological conditions involving E/I disruption, such as epilepsy and autism spectrum disorder.

The major underlying pathological mechanism in epilepsy is an imbalance between glutamatergic excitation and γ-aminobutyric acid (GABA)-ergic inhibition (21–24), which results in uncontrolled synchronous neuronal excitation (25, 26). Furthermore, unregulated firing of neurons is also observed in autism spectrum disorder, and indeed there is high comorbidity between epilepsy and autism spectrum disorder (27). Therefore, diagnosis and prognosis may benefit from measurement of general glutamatergic and GABAergic function. Although magnetic resonance spectroscopy has been applied to measure the GABA concentration in the human brain, results are often ambiguous and dependent on the scanning sequence (28, 29). Alternatively, E/I balance in the cortex may be inferred from psychophysical tasks. For instance, BR is clearly linked to cortical E/I balance (30–32), Freyberg et al., 2015, (33, 34). Moreover, forms of epilepsy classified as idiopathic generalized epilepsy (IGE) are disorders of presumed genetic origin without macroscopic brain abnormalities (35). Therefore, BR may be an ideal task for indirect assessment of E/I balance in IGE.

Epileptiform discharges can be viewed as an eruption of excess excitatory drive (36) that is reflected by a higher frequency and amplitude waveform on the electroencephalogram (EEG) (37, 38). The EEG of IGE patients features symmetrical waves starting at a focus and rapidly spreading to bilateral brain networks (39). Further, IGE patients may also exhibit abnormal waveforms in the absence of seizures, termed interictal epileptiform discharges (IEDs) that are frequently measured by ambulatory EEG.

As the balance between γ-GABAergic inhibitory mechanisms and glutamatergic excitatory mechanisms underlies both IGE and BR, BR may be altered in IGE and thus serve as a useful diagnostic or prognostic marker. It is therefore of interest to explore the features of BR in IGE patients and the associations between BR changes and relevant clinical factors.

## Materials and Methods

### Participants

Ninety-four patients with IGE (48 males, 46 females) were enrolled from the Department of Neurology, First Affiliated Hospital of Anhui Medical University, Hefei, Anhui Province, China. Sixty-two healthy control (HC) subjects (28 males, 34 females) were also enrolled, all students or social personnel. IGE and HC subjects were matched for age, gender, and years of education. All patients were clinically diagnosed with IGE based on seizure history, ambulatory EEG, and neuroimaging as defined by the Commission on Classification and Terminology of the International League Against Epilepsy (40). IGE is a group of clinical syndromes, including benign familial neonatal epilepsy, benign infant epilepsy, benign familial infantile epilepsy, childhood absence epilepsy, juvenile absence epilepsy, juvenile myoclonic epilepsy, and generalized tonic-clonic epilepsy, belonging to the hereditary generalized epilepsy. This cohort included patients with four distinct IGE syndromes, childhood absence epilepsy, juvenile absence epilepsy, juvenile myoclonic epilepsy, and generalized tonic-clonic epilepsy. All patients received 24-h ambulatory EEG monitoring from 2:30 to 2:30 p.m. the next day. During the recording, normal living rhythm was maintained, including normal sleeping and awaking cycles. Patients were stratified into abnormal ambulatory EEG (AB-AEEG) and normal ambulatory EEG (N-AEEG) subgroups depending on the presence or absence, respectively, of IEDs on the ambulatory EEG. We recorded the number and total duration of IEDs in IGE patients with abnormal ambulatory EEG. All participants were free of color blindness, poor visual acuity, strabismus, and other visual impairments. All participants were right-handed and had the same eye dominance. All participants also demonstrated normal cognitive function, with sufficient oral, visual, and written language skills to complete the experimental tasks. Participants did not ingest coffee, tea, cola, or alcohol for at least 4 h before testing as these beverages may affect BR (41, 42).

Exclusion criteria were (1) history of cerebral infarction, space-occupying intracranial lesions, infection, or brain trauma; (2) intracranial disease as evidenced by computed tomography or magnetic resonance imaging; (3) long-term use of medications other than antiseizure medicines (ASMs); (4) history of epilepsy correction surgery; (5) current psychiatric conditions such as moderately severe anxiety, depression, and history of drug abuse; or (6) a history of severe chronic physical illness.

During the experiment, all IGE patients were in the interictal state, and all participants were naive to the purpose of the study. Finally, 94 patients with IGE were enrolled, 64 with abnormal ambulatory EEG (AB-AEEG group) as defined by the presence of interictal discharges and 30 with normal ambulatory EEG (N-AEEG group). The 64 patients with abnormal AEEG included 23 with no ASM history and 41 who had received ASMs. Twenty-three patients were new-onset cases with no ASM history, while all others had demonstrated good ASM adherence with no seizures for at least 3 months. Detailed information about ASMs is presented in (Supplementary Table 1). Some IGE patients also had mild anxiety or depression, but were not receiving anxiolytic or antidepressant drugs. Written informed consent was obtained from all participants and the study was approved by the Anhui Medical University Ethics Committee.

### Psychometric Testing

All participants completed the Montreal Cognitive Assessment (MoCA) to confirm normal cognition as well as the Beck Depression Inventory (BDI) and Hamilton Anxiety Scale (HAMA) to assess current depression and anxiety symptoms. The BR test was performed within 2 h before 24 h ambulatory EEG monitoring. All assessments were conducted by the same trained assessor.

### BR Task

The BR task was conducted in a dimly lit closed room. Lenovo laptop (15.6-inch monitor screen, 1,366 × 768 resolution, 60 Hz refresh rate) was used to display the visual stimuli. Stimulus presentation and trial timing were controlled by Psychtoolbox running in MATLAB (8, 9). Subjects sat about 100 cm from the screen and wore red-green stereoscopic glasses while viewing overlapping color-filtered images of a green radial raster presented to the left eye (four cycles/deg) and a red concentric circular raster to the right eye (eight cycles/deg) to induce BR. Overall image luminance was 135 cd/m^2^, sine wave 0.9, background luminance 30 cd/m^2^, and black frame was 1.6° × 1.6°.

To ensure participants understood the BR task, they received instructions and a practice session before starting the actual BR trials. Subjects were instructed to focus on the center of the screen and press a right response button when the green radial raster image was perceptually predominant and a left response button when the red concentric circular raster image was predominant. Following the practice session, three BR trials were conducted, each lasting 120 s, with an inter-trial interval of 3 min.

In each test session, subjects also completed three pseudo-randomly programmed catch trials to ensure accurate performance of the binocular rivalry task. In the catch trials, two monocular stimuli were alternately presented separately, with each stimulus between 0.5 and 5 s, to roughly simulate the transformation in perception experienced during rivalry. The response was considered correct only when the subject pressed the correct button in response to the stimulus within the time window of 800 ms. To verify that the subjects understood and accurately completed the task, the average correct response rate of the catch trials was recorded.

Perceptual rivalry rate was counted as the number of rivalry conversions (right to left button transitions and vice versa) divided by the total viewing time (in seconds). Participants were instructed to view all stimuli passively, without attempting to control their perceptions. The behavioral indicator of binocular rivalry was the alternation rate (in Hz).

### Data Analyses

Numeric variables are presented as mean ± standard deviation or median [25^th^-75^th^ quartile], and nominal variables as numbers and percentages. Group means were compared by independent samples *t*-tests or one-way ANOVA in the case of two or more than two groups, respectively. The chi-square test was used to compare ratios among groups. The non-parametric Mann-Whitney U test was used to compare the BDI score and HAMA score between two groups, and non-parametric test (Kruskal-Wallis H) was used to compare the BDI score and HAMA score among the three groups, expressed as M (P25, P75). Pearson and Spearman correlation analyses were used to examine within-subject reliability for the perceptual alternation rate and the associations between alternation rate and various demographic and clinical characteristics. In the correlation analysis, Pearson or Spearman correlation analysis was selected according to whether the two related variables followed a normal distribution. Multivariable linear regression analysis was used to explore the effects of demographic and clinical characteristics (such as age, gender, education level, MoCA score, HAMA score, BDI score, ambulatory EEG-related variables, and ASM-related variables) on the alternation rate. The ambulatory EEG-related variables were the number of IEDs, total duration of IEDs, and presence or absence of IEDs. The medication-related variable was the ASM used (namely, valproic acid, lamotrigine, carbamazepine, oxcarbazepine, levetiracetam, phenytoin, or phenobarbital).

Multivariable linear regression analysis was used, with *p* > 0.1 as the exit criterion and *p* < 0.05 as the entry criterion, to evaluate the factors correlated with the alternation rate. The dependent variable was the BR alternation rate. The independent variables included age, gender, education level, MoCA score, HAMA score, BDI score, ambulatory EEG-related variables, and ASM-related variables. Receiver operating characteristic (ROC) curves of the BR alternation rate, MoCA score, and the two variables combined were analyzed, and the area under the curve (AUC) was calculated with 95% confidence intervals (95% CIs) to assess specificity, sensitivity, Youden's index (YI), and cut-off. To further evaluate the predictive performance of BR alternation rate and MoCA, we constructed a multivariable linear regression model combined with k-fold cross-validation (43). The BR alternation rate, the MoCA score and the combination of the two variables were analyzed by using the model. In the model, the value of k is set to 3. In 3-fold cross-validation, the date set is split into 3-folds and the method is repeatedly performed 3 times. Each time one of the k folds is used for the test. A model is trained using k-1 of the folds as training sets (44). The ROC response of different training sets is obtained from the trained model. Performance of the prediction model was evaluated as the average area under the ROC curve (AUC), sensitivity, and specificity across all k trains are then computed. A *p* < 0.05 (2 tailed) was considered significant for all tests. All statistical analyses were conducted using Statistical Package for the Social Sciences (SPSS) version 26.0 (International Business Machines Corp., Armonk, NY, USA).

## Results

### Subject Characterization

**Figure 1:**
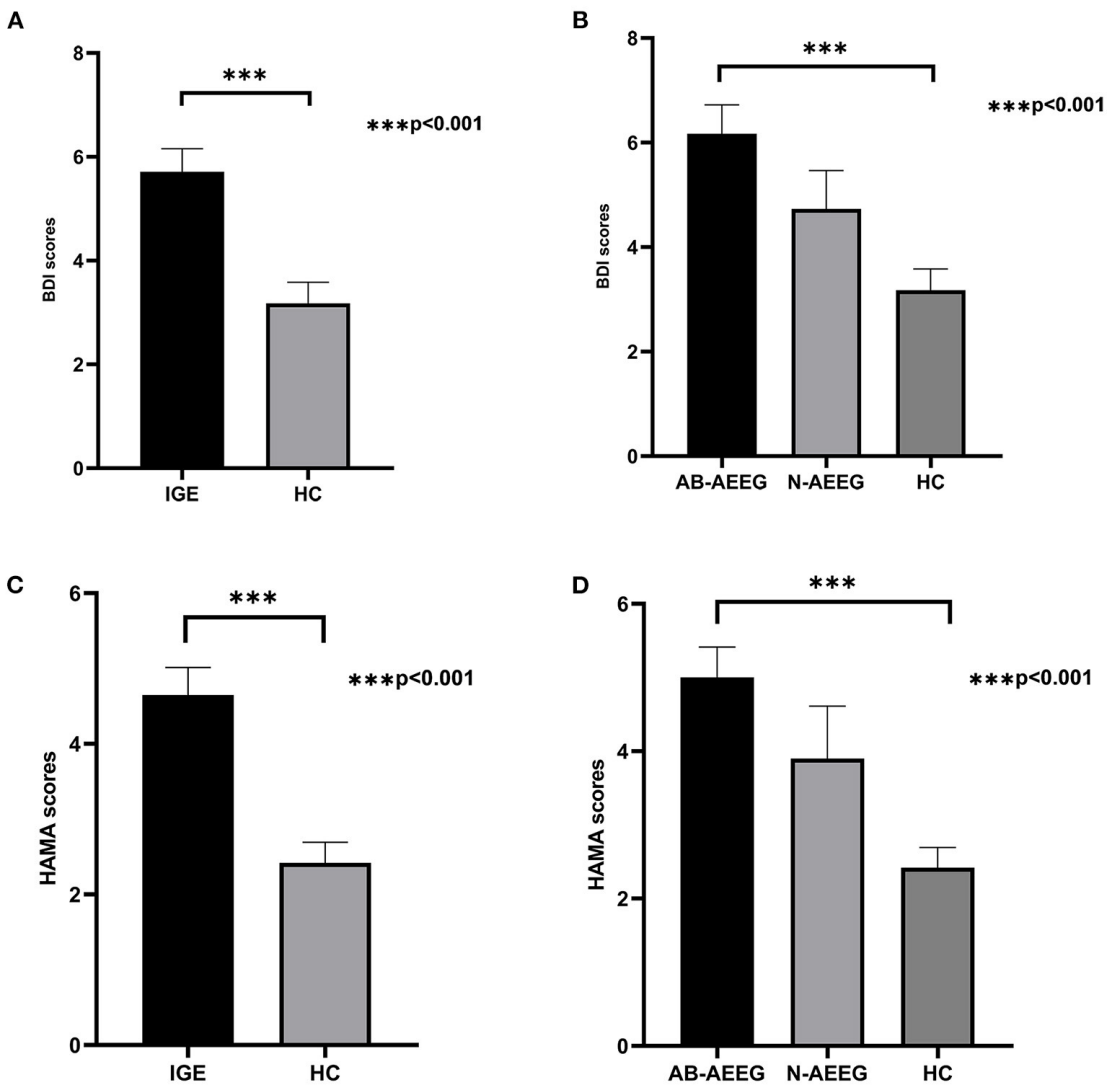
Differences in BDI and HAMA scores among groups. IGE, idiopathic generalized epilepsy; AB-AEEG, abnormal ambulatory electroencephalogram; N-AEEG, normal ambulatory electroencephalogram; HC, healthy control; BDI, Beck Depression Inventory; HAMA, Hamilton Anxiety Scale. **(A)** BDI score was higher in the IGE group than HC group (*Z* = −3.773, *p* < 0.001). **(B)** BDI scores of the three groups (AB-AEEG, N-AEEG, and HC). BDI score was higher in the AB-AEEG group than HC group (χ^2^ = 16.492, *p* < 0.001). **(C)** HAMA score was higher in the IGE group than HC group (*Z* = −4.109, *p* < 0.001). **(D)** HAMA scores of the three groups, The HAMA score was significantly higher in the AB-AEEG group than HC group (χ^2^ = 21.214, *p* < 0.001).

The demographic and clinical characteristics of the total IGE group, the two IED-stratified IGE subgroups (AB-AEEG and N-AEEG), and the HC group are summarized in Table 1. There were no significant differences in age, gender, years of education, and MoCA score. As expected, there were significant differences in BDI and HAMA scores between the total IGE group and HC group (*Z* = −3.773, *p* < 0.001; *Z* = −4.109, *p* < 0.001, respectively). BDI and MAHA scores also differed significantly among the HC, AB-AEEG, and N-AEEG groups (χ^2^ = 16.492, *p* < 0.001; χ^2^ = 21.214, *p* < 0.001, respectively). *Post-hoc* pair-wise comparisons revealed that BDI score was significantly higher in the AB-AEEG group than the HC group (*p* < 0.001) but did not differ between the N-AEEG and HC groups (*p* = 0.220), or the N-AEEG and AB-AEEG groups (*p* = 0.287). The HAMA score was also significantly higher in the AB-AEEG group than the HC group (*p* < 0.001) but did not differ between the N-AEEG and HC groups (*p* = 0.090), or the N-AEEG and AB-AEEG groups (*p* = 0.312) (Table 1, Figure 1).

**Table 1:** Demographic and clinical characteristics of the participants.

**Item**	**IGE**	**IGE**		**HC**
	**(*n* = 94)**	**AB-AEEG**	**N-AEEG**	**(*n* = 62)**
		**(*n* = 64)**	**(*n* = 30)**	
Age (years), mean ± SD	18.81 ± 5.61	18.31 ± 5.61	19.87 ± 5.54	19.19 ± 4.41
(range)	(10–37)	(10–37)	(10–33)	(12–30)
Female, n (%)	46 (48.94%)^*^	33 (51.56%)^*^	13 (43.33%)^*^	34 (54.84%)^*^
Education (years), mean ± SD	8.96 ± 2.81	8.63 ± 2.43	9.68 ± 3.41	9.27 ± 2.21
BDI, *M* (P25, P75)	5.00 (2.75, 9.00)^a^	6.00 (3.00, 9.00)^b^	5.00 (1.00, 6.25)	2.50 (0.00, 5.00)^a^^b^
HAMA, *M* (P25, P75)	4.00 (2.00, 7.00)^a^	5.00 (2.00, 7.00)^b^	3.50 (1.00, 5.50)	2.00 (0.00, 3.00)^a^^b^
MoCA, mean ± SD	25.39 ± 5.44	25.23 ± 2.42	25.73 ± 2.50	26.06 ± 1.96

* *Chi-square test (χ^2^ = 0.521, p = 0.470; (χ^2^ = 1.075, p = 0.584)*.

a *p < 0.001*,

b *p < 0.001. IGE, idiopathic generalized epilepsy; AB-AEEG, abnormal ambulatory electroencephalogram; N-AEEG, normal ambulatory electroencephalogram; HC, healthy control; BDI, Beck Depression Inventory; HAMA, Hamilton Anxiety Scale; MoCA, Montreal Cognitive Assessment; SD, standard deviation; M, median; P25, 25^th^ percentile; P75, 75^th^ percentile*.

### Accuracy of the BR Test

Univariate correlation analysis showed that the BR alternation rate between trial blocks 1 and 3 were well correlated within groups (IGE: *r* = 0.781, *p* < 0.001; AB-AEEG: *r* = 0.768, *p* < 0.001; N-AEEG: *r* = 0.805 *p* < 0.001; HC: *r* = 0.804, *p* = *p* < 0.001), indicating good within-subject consistency. The alternation rate accuracies in catch trials were above 95% in all groups (IGE: 98.51 ± 4.17; AB-AEEG: 98.46 ± 4.48; N-AEEG: 98.64 ± 5.06; HC: 98.93 ± 3.78), indicating high task reliability. No significant differences in catch trial accuracy were found among the IGE, AB-AEEG, N-AEEG, and HC groups (Table 2).

**Table 2:** BR alternation rates of different IGE groups and subgroups.

**Item**	**IGE**	**IGE**		**HC**
	**(*n* = 94)**	**AB-AEEG**	**N-AEEG**	**(*n* = 62)**
		**(*n* = 64)**	**(*n* = 30)**	
Catch trial accuracy (%), mean ± SD	98.51 ± 4.71	98.46 ± 4.48	98.64 ± 5.06	98.93 ± 3.78
Trial 1.vs. Trial 3, r	0.781^*^	0.768^*^	0.805^*^	0.804^*^
Mean rivalry rate (Hz), mean ± SD	0.34 ± 0.13^c^	0.28 ± 0.07^d^^e^	0.46 ± 0.14^e^	0.43 ± 0.10^c^^d^
Median rivalry rate (Hz), mean ± SD	0.31 ± 0.01^c^	0.27 ± 0.01^d^^e^	0.44 ± 0.03^e^	0.40 ± 0.01^c^^d^
Mode (Hz), mean ± SD	0.15 ± 0.01	0.15 ± 0.01	0.23 ± 0.03	0.24 ± 0.01
Variance (Hz), mean ± SD	0.017 ± 0.01	0.005 ± 0.01	0.027 ± 0.03	0.011 ± 0.01

* *Pearson correlation analysis*.

c *p < 0.001*,

d *p < 0.001*,

e *p < 0.001. IGE, idiopathic generalized epilepsy; AB-AEEG, abnormal ambulatory electroencephalogram; N-AEEG, normal ambulatory electroencephalogram; HC, healthy control; SD, standard deviation*.

### Mean BR Alternation Rates

**Figure 2:**
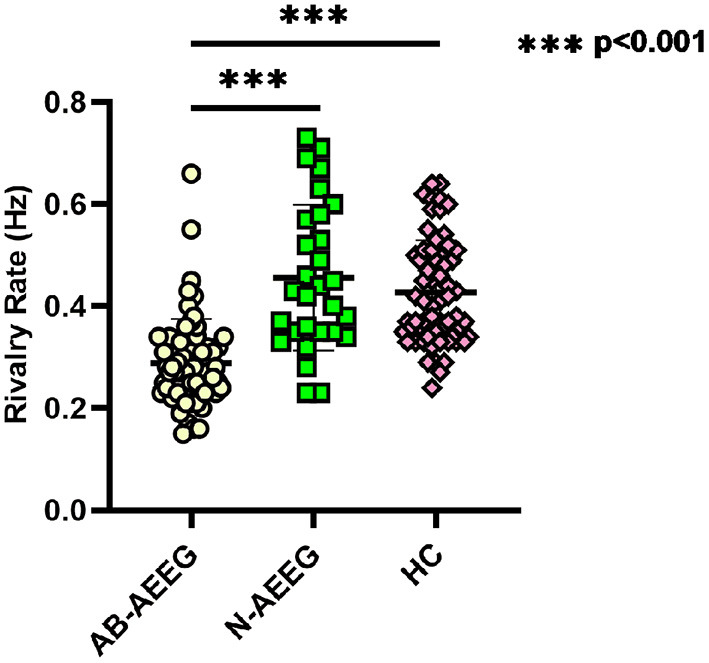
Scatter diagram of rivalry rates for AB-AEEG, N-AEEG and HC. AB-AEEG, abnormal ambulatory electroencephalogram; N-AEEG, normal ambulatory electroencephalogram; HC, healthy control; One-way ANOVA demonstrated a statistical difference in alternation rate among the HC, AB-AEEG, and N-AEEG groups (*F* = 44.962, *p* = 0.000). BR alternation rate was significantly slower in the AB-AEEG group compared to the N-AEEG and HC groups (0.28 vs. 0.46 Hz, *p* < 0.001; 0.28 vs. 0.43 Hz, *p* < 0.001). There was no difference between N-AEEG and HC groups (*p* = 0.348). ***p < 0.001.

The BR alternation rate was significantly lower in the IGE group compared with the HC group (0.34 ± 0.13 vs. 0.43 ± 0.10, *t* = −4.364, *p* < 0.001) (Table 2). One-way ANOVA also revealed a significant difference in alternation rate among the HC, AB-AEEG, and N-AEEG groups (*F* = 44.962, *df* = 2, *p* < 0.001). Furthermore, the Bonferroni corrected *t*-test revealed that the BR alternation rate was significantly slower in the AB-AEEG group compared with that in the N-AEEG and HC groups (0.28 vs. 0.46 Hz, *p* < 0.001; 0.28 vs. 0.43 Hz, *p* < 0.001) but did not differ between the N-AEEG and HC groups (0.46 ± 0.14 vs. 0.43 ± 0.10, *p* = 0.348) (Table 2, Figure 2).

### Correlations Between BR Alternation Rate and Clinicodemographic Characteristics in IGE

To identify demographic and clinical characteristics influencing the BR alternation rate, we first conducted univariate analyses among IGE patients. Both the AB-AEEG and MoCA scores were significantly correlated with the alternation rate in IGE patients (*r* = 0.584, *p* < 0.001; *r* = 0.298, *p* = 0.004, respectively), while there were no significant correlations between the alternation rate and age, gender, education level, ASM-related variables, BDI score, and HAMA score (Tables 3, 4).

**Table 3:** Correlations of demographic and clinical characteristics with rivalry alternation rate in the IGE group (*n* = 94).

	**Pearson correlation**	**Age**	**Gender**	**Years of education**	**AEEG**	**MoCA**	**BDI**	**HAMA**
Rivalry rate (Hz)	r	0.009	−0.072	0.133	0.584^***^	0.298^**^	−0.127	−0.175
	*p*	0.928	0.487	0.200	0.000	0.004	0.221	0.092

*** *P < 0.001*.

** *p < 0.01. AEEG, ambulatory electroencephalogram; MoCA, Montreal Cognitive Assessment; BDI, Beck Depression Inventory; HAMA, Hamilton Anxiety Scale*.

**Table 4:** Summary of AEEG status, ASMs use, and correlations with rivalry alternation rates in IGE patients.

	**Mean (SD) or r**	***p-*value**
ASMs treatment	−0.143	0.234
Monotherapy (*n* = 51)	0.380 (0.152)	
Polytherapy (*n* = 20)	0.325 (0.098)	
Use of valproic acid		0.755
Yes (*n* = 26)	0.376 (0.149)	
No (*n* = 45)	0.359 (0.136)	
Use of lamotrigine		0.480
Yes (*n* = 13)	0.384 (0.139)	
No (*n* = 58)	0.362 (0.142)	
Use of Levetiracetam		0.727
Yes (*n* = 2)	0.312 (0.037)	
No (*n* = 69)	0.367 (0.142)	
Use of Carbamazepine		0.680
Yes (*n* = 4)	0.366 (0.189)	
No (*n* = 67)	0.366 (0.137)	
Use of Oxcarbazepine		0.181
Yes (*n* = 4)	0.288 (0.113)	
No (*n* = 67)	0.372 (0.142)	
Other ASMs (*n* = 3)		
AEEG	0.584	0.000^***^
Normal (*n* = 30)	0.455 (0.142)	
Abnormal (*n* = 64)	0.288 (0.886)	

*** *There was significant correlation between AEEG with rivalry rates, P < 0.001. ASMs, antiseizure medicines; AEEG, ambulatory electroencephalogram*.

### Factors Independently Associated With Binocular Rivalry Alternation Rate in IGE

A stepwise linear regression model (including age, gender, education level, MoCA score, HAMA score, BDI score, ambulatory EEG-related variables, and ASM-related variables) demonstrated AEEG status [β, 0.173; standard error (SE), 0.022; *p* < 0.001] and MoCA score (β, 0.013; SE, 0.004; *p* = 0.003) as independent factors associated with the BR alternation rate (Table 5). These models for ambulatory EEG and the MoCA score explained 41.0% and 8.9% of the variance in alternation rates, respectively.

**Table 5:** Stepwise linear regression showing factors independently associated with rivalry rate in patients with IGE.

	**The alternation rates of binocular rivalry (*****n*** **=** **94)**				
	**β**	**SE**	**beta**	**VIF**	***P*-value**
AEEG	0.173	0.022	0.617	1.009	0.000^***^
MoCA	0.013	0.004	0.239	1.009	0.003^**^

*** *p < 0.001*.

** *p < 0.01. AEEG, ambulatory electroencephalogram; MoCA, Montreal Cognitive Assessment; β, non-standardized coefficient; SE, standard error; beta, standardized coefficient; VIF, variance inflation factor*.

### Discrimination Power of BR Alternation Rate and MoCA Score in IGE

**Figure 3:**
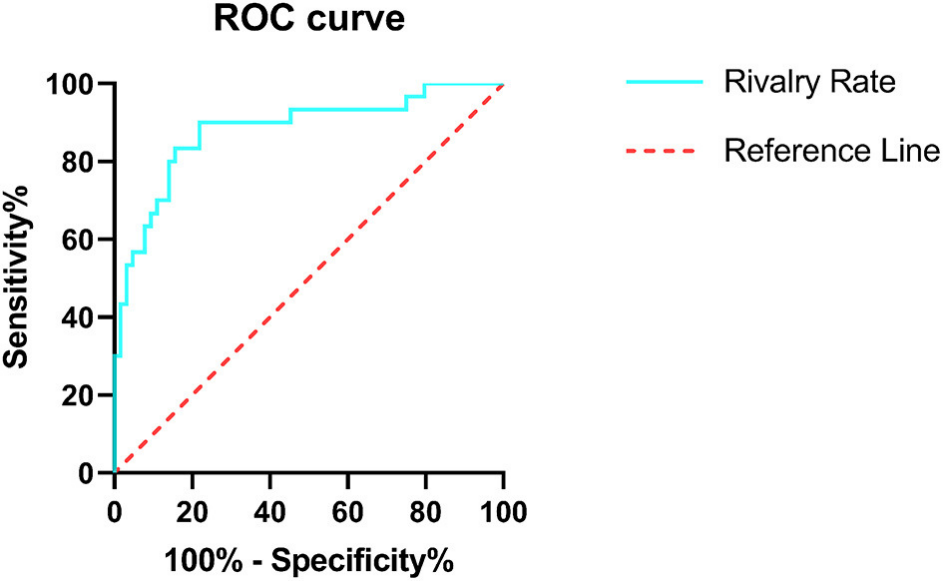
Receiver operating characteristic (ROC) curve of the BR rivalry rate. ROC analysis was used to assess the diagnostic efficiency. The area under the curve (AUC) was 0.881 (95% CI = 0.801–0.961), sensitivity 90.00%, specificity 76.90%, and YI was 0.681. The ROC curve is shown in green and the reference line in red.

**Figure 4:**
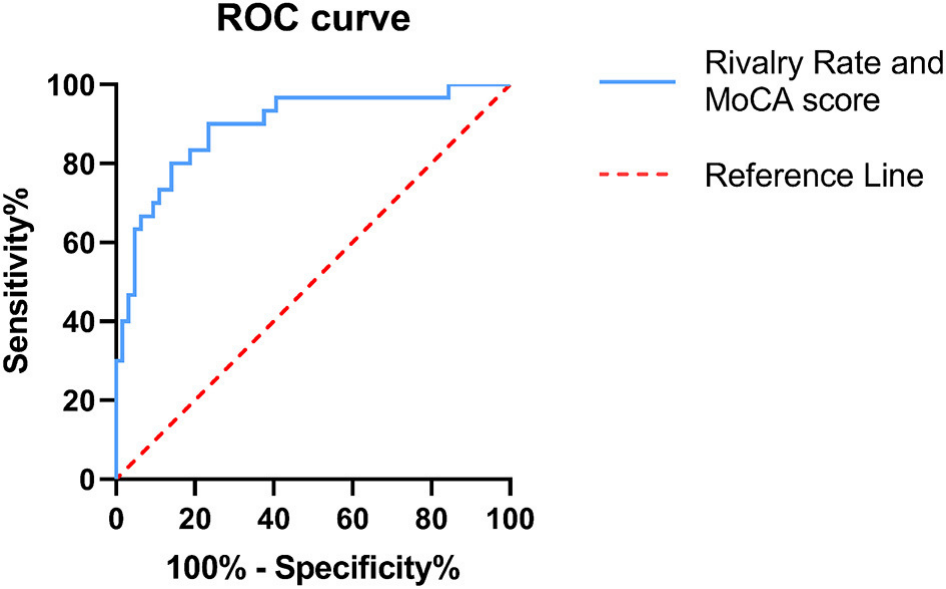
Receiver operating characteristic (ROC) curve combining both the BR alternation rate and MoCA score. ROC analysis was used to assess the diagnostic efficiency. The area under the curve (AUC) was 0.894 (95% CI = 0.882–0.966). The ROC curve is shown in blue and the reference line in red.

**Figure 5:**
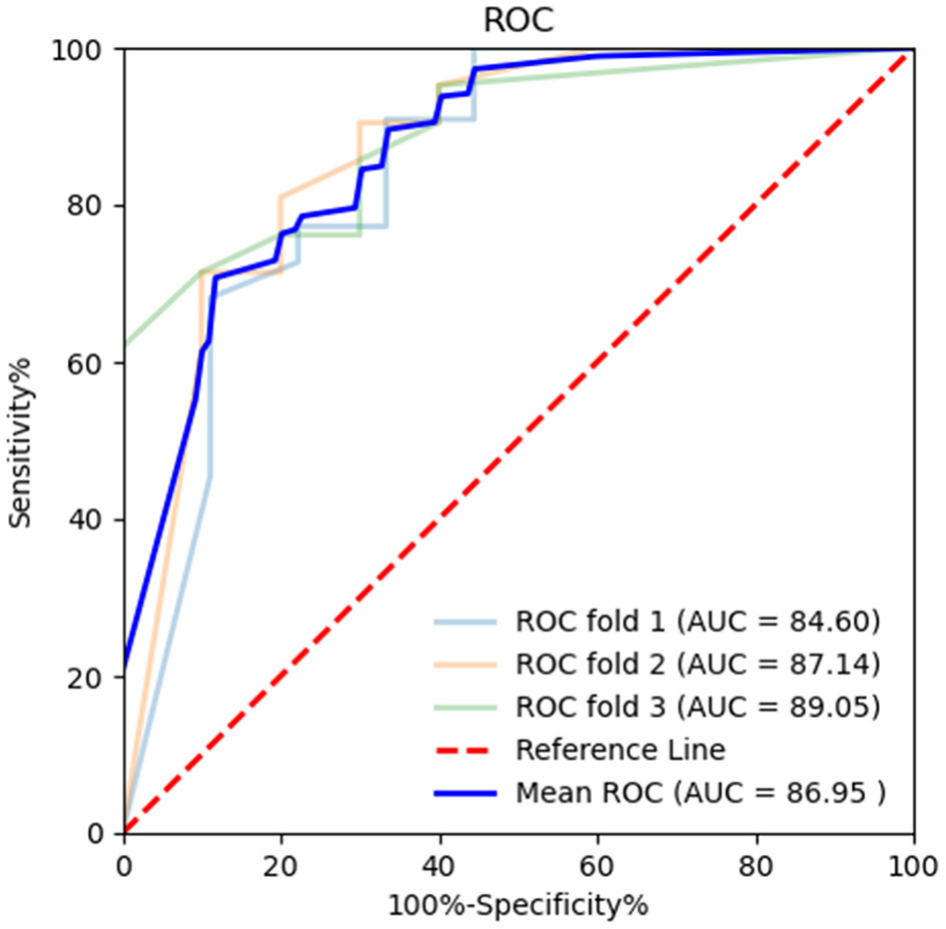
Receiver operating characteristic (ROC) curve of BR alternation rate in k-fold cross-validation. K-fold cross-validation was used to evaluate the predictive performance of BR alternation rate and MoCA. The average area under the curve (AUC) was 0.870, sensitivity 89.36%, and specificity 62.25%.

**Figure 6:**
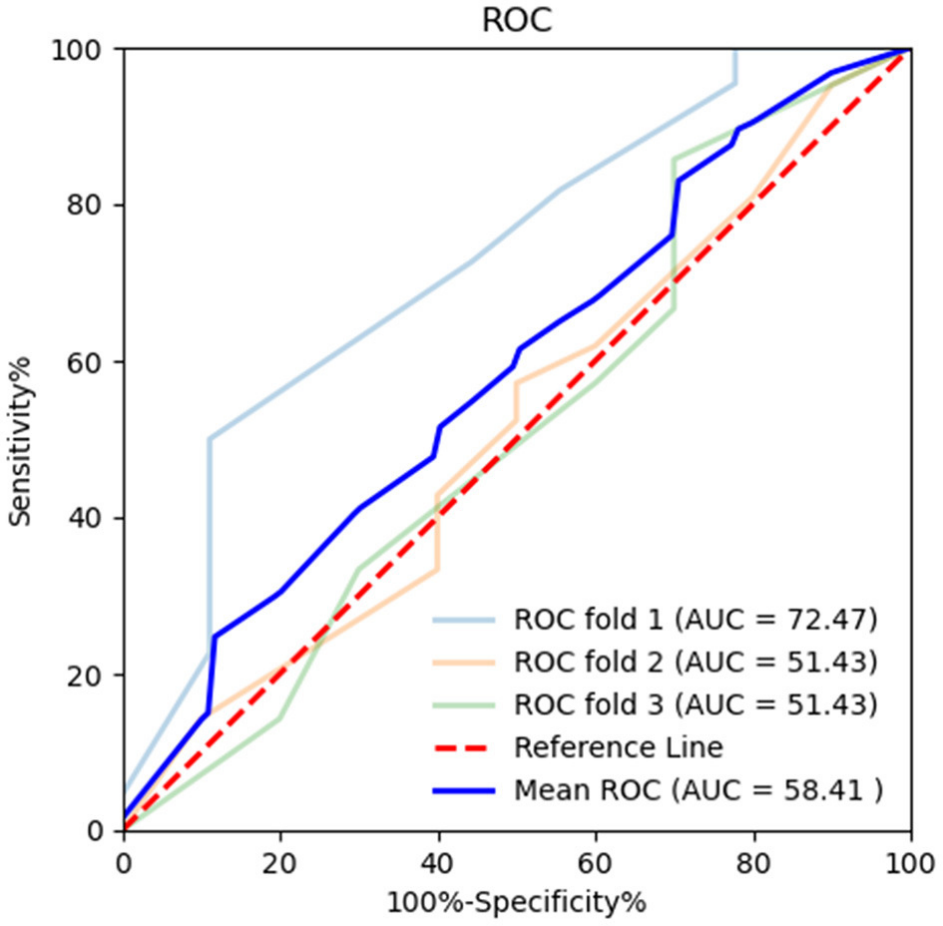
Receiver operating characteristic (ROC) curve of MoCA in k-fold cross-validation. K-fold cross-validation was used to evaluate the predictive performance of MoCA. The mean area under the curve (AUC) was 0.584, sensitivity 92.73%, and specificity 13.42%.

**Figure 7:**
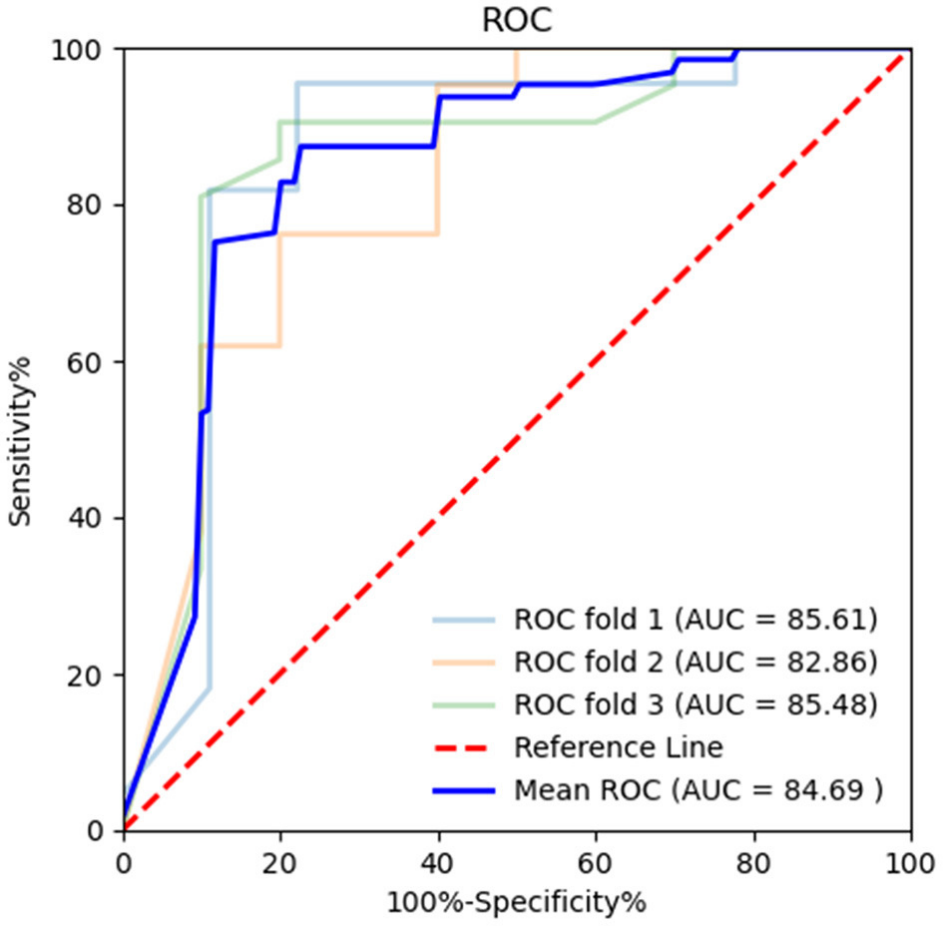
Receiver operating characteristic (ROC) curve combining both the BR alternation rate and MoCA score in k-fold cross-validation. K-fold cross-validation was used to evaluate the predictive performance. The mean area under the curve (AUC) was 0.847, sensitivity 91.28%, and specificity 61.78%.

Diagnostic efficiency of the BR alternation rate and MoCA score was evaluated using ROC analysis, which yielded AUC values of 0.881 (95% CI = 0.801–0.961, *p* < 0.001) and 0.566 (95% CI = 0.441–0.691, *p* = 0.307), respectively. The ROC curve of the BR alternation rate distinguished AB-EEG from N-AEEG subjects with 90.00% sensitivity, 76.90% specificity, and YI of 0.681 at a cut-off of 0.391 Hz (Figure 3). At this cut-off, the false-positive rate (N-AEEG identified as AB-AEEG) was 9.68%. The ROC curve of the MoCA score yielded 43.30% sensitivity, 67.20% specificity, and a YI of 0.105 at a cut-off score of 26.5. The ROC curve combining both the BR alternation rate and MoCA score yielded an AUC value of 0.894 (95% CI = 0.822–0.966, *p* < 0.001) (Figure 4). We also evaluated the predictive performance of BR alternation rate, MoCA score, and the combination of both the two variables using k-fold cross-validation, which yielded average AUC values of 0.870, 0.584 and 0.847, average sensitivity values of 89.36%, 92.73% and 91.28%, and average specificity values of 62.25, 13.42, and 61.78%, respectively (Figures 5–7).

### Detection of IEDs From Ambulatory EEG and the Correlation Between the IEDs Burden and BR Rate

We also determined the number and total duration of IEDs in IGE patients with epileptiform discharges to assess the potential relationship with BR rate. The median of the number of IEDs was 9.50 [4.00–17.75] and the median of the total duration of IEDs was 9.80 s [4.90–24.9 s]. To examine if the IED burden influenced the BR alternation rate, we conducted Pearson correlation analyses. Indeed, the number of IEDs was significantly correlated with the alternation rate in IGE patients (*r* = 0.296, *p* = 0.018), while there was no significant correlation between the alternation rate and total duration of IEDs (*r* = 0.097, *p* = 0.445). To examine whether the number of IEDs is independently associated with the BR rate, we constructed a forward stepwise linear regression model (including the total duration of IEDs, age, gender, education level, MoCA score, HAMA score, and BDI score). The results demonstrated that the number of IEDs [β, 0.001; standard error (SE), 0.001; *p* = 0.025] is an independent factor influencing BR alternation rate in patients with IEDs (Table 6). This model explained 18.6% of the variance in BR alternation rates.

**Table 6:** Forward stepwise linear regression showing factors independently associated with rivalry rate in patients with IEDs.

	**The alternation rates of binocular rivalry (*****n*** **=** **64)**				
	**β**	**SE**	**beta**	**VIF**	***P*-value**
IEDs number	0.001	0.001	0.361	1.651	0.025^*^

* *P < 0.05. IEDs, interictal epileptiform discharges; β, non-standardized coefficient; SE, standard error; beta, standardized coefficient; VIF, variance inflation factor*.

## Discussion

The clinical significance of bistable perceptual dynamics is well documented, including numerous studies reporting abnormal bistable perception among subjects with pathological conditions compared with healthy subjects (30), Freyberg et al., 2015, (45–49). In the current study, we demonstrated that patients with IGE exhibit slower alternation rates than HCs during a BR task. In other words, bistable perceptual alternation rates during BR are slower in IGE patients, which may reflect abnormalities in cortical dynamics. However, this abnormality was specific to patients with IEDs, as patients without such discharges on the ambulatory EEG (the N-AEEG subgroup) exhibited BR alternation rates roughly equivalent to HCs. These results indicate that IEDs transiently disrupt the neural processes mediating perceptual switching in the BR task.

We then assessed whether various clinical and demographic factors influence the BR alternation rate. Spearman correlation analysis indicated that the use of individual ASMs has no effect, nor did multiple demographic factors, further underscoring the importance of IEDs for BR. We also explored this influence by stepwise linear regression, after adjusting for potential confounding factors (including age, gender, education level, MoCA score, HAMA score, BDI score, and ASM-related variables), which confirmed that IEDs on the ambulatory EEG is an independent predictor of slower BR alternation rate. ROC analysis showed that a slow BR alternation rate predicted the presence of an abnormal ambulatory EEG with an AUC value of 0.881 and 90.00% sensitivity and 76.90% specificity. We also evaluated the diagnostic efficiency of the MoCA score using ROC, which yielded an AUC value of 0.566, and *p* > 0.05, indicating that the MoCA score alone has little predictive power. Further, the ROC curve combining the BR alternation rate and MoCA score yielded an AUC value only slightly higher than the BR alternation rate alone (0.894). These results indicate that BR alternation rate can be used to distinguish IGE cases complicated with IEDs. Similarly, Pitts et al. (50) demonstrated that EEG source imaging provides high temporal resolution and whole-brain spatial coverage for binocular rivalry. Therefore, BR could be a valuable auxiliary behavioral tool to diagnose and dynamically monitor disease status in IGE patients. The mechanisms underlying the influence of IEDs on BR alternation rate require further investigation.

To examine the relationships between BR alternation rate and both the number and total duration of IEDs, we conducted Pearson correlation analyses and found that the number of IEDs was significantly associated with alternation rate in IGE patients, while there was no correlation between alternation rate and total duration of IEDs. Moreover, a forward stepwise linear regression model adjusting for potential confounding factors (including the total duration of IEDs, age, gender, education level, MoCA score, HAMA score, and BDI score) confirmed that the number of IEDs was an independent factor associated with the BR rate in patients with IEDs.

There are several possible mechanisms for the slower rate of binocular rivalry in IGE patients with higher IED frequency. First, interictal discharges may transiently disrupt perceptual dynamics, thus slowing the rate of BR. Tong et al. (2) proposed a hybrid model based on previous research that may account for the paradoxical effects of inhibitory and excitatory circuits on BR and even provides insights into the neural bases of visual awareness itself. Brain imaging studies have also indicated that bistable perception is associated with shifting neural dynamics within both early visual areas (6) and higher centers such as the frontal and parietal cortex (7–11). Epileptiform discharges can cause an imbalance in the cortex (21–24). Our research confirmed that a greater number of IEDs (but not longer duration) independently reduces the BR rate, suggesting that the transient cortical imbalance caused by interictal discharges may interfere with bistable perception.

Second, IEDs may cause an acute decline in cognitive function (51–53), which in turn slows the BR rate. The MoCA score in the AB-AEEG group was slightly lower than in the N-AEEG and HC groups, suggesting that interictal discharges impair cognitive functions related to bistable perception. Moreover, univariate analyses revealed that the MoCA score was significantly correlated with the BR rate in IGE patients, and stepwise linear regression revealed that the MoCA score was an independent predictor of the BR alternation rate after adjusting for potential confounding factors. However, there is disagreement about whether cognitive function influences the BR alternation rate. Zhang et al. (54) posited that attention may affect alternation rate, but Vierck et al. (55) reported that the BR alternation rate was independent of cognitive functions such as memory and attention. Stepwise linear regression identified also independent influences of AEEG status and MoCA score on the BR alternation rate. While MoCA score explained only 8.9% of the variance, AEEG score explained 41.0%. Furthermore, ROC analysis revealed that the BR alternation rate can distinguish IGE patients with and without IEDs, while the MoCA score has little predictive power. Our results suggest that the influence of cognitive capacity on the BR alternation rate is statistically significant but limited.

Third, the ASMs taken by these patients may affect BR rate indirectly by disrupting cognitive function. ASMs are known to slow cognitive function, including valproic acid (56), benzodiazepines, and phenobarbital (57). As patients were on different medications, the influences of ASM type and dose cannot be ruled out. The effects of ASMs on binocular rivalry are complex because of the variety of mechanisms underlying ASM clinical efficacy (58). Due to the sample size, the influences of different ASMs on BR rate could not be evaluated. In future studies, the impact of different drug types and dosages on binocular rivalry could be explored by increasing the sample size and stratifying subgroups according to types and dosages of ASMs.

Fourth, IEDs may disrupt excitatory circuits, inhibitory circuits, or both required for perceptual switching. Epileptic seizures arise from an imbalance between glutamatergic excitation and GABAergic inhibition (21–24). Binocular rivalry is linked to cortical E/I balance (30–32), Freyberg et al., 2015, (33, 34). Thus, we speculate that IEDs may disrupt the balance between glutamatergic excitation and GABAergic inhibition in the cortex, thereby affecting the BR rate of IGE patients. However, the mechanism of cortical E/I imbalance in epileptic seizures is based on animal models and has not been verified in humans with IGE. Further, different epilepsy syndromes, may arise through distinct pathogenic mechanisms. Thus, the effects of interictal discharge frequency on BR in IGE may be highly patient-specific. A possible alternative mechanism is that interictal discharges may transiently disrupt perceptual dynamics or cognition, thus slowing the rate of BR, and different epilepsy syndromes may have different mechanisms.

Anxiety and depression are common psychiatric comorbidities in epilepsy, with incidence rates far exceeding those of the general population (59–62). According to epidemiological estimates, as many as 25% of epilepsy patients suffer from anxiety (59). In our study, IGE patients obtained higher HAMA scores than HCs, and the AB-AEEG group obtained higher HAMA scores than the N-AEEG and HC groups. A systematic review and meta-analysis also found active depression in 23.1% of epilepsy patients, about 4–5 times higher than in the general population (63). We also found higher depression (BDI) scores among IGE patients, and higher scores among the AB-AEEG group than the N-AEEG and HC groups. Thus, IEDs may be associated with greater prevalence of depression and anxiety.

Previous studies have suggested that BR alternation rate may be influenced by participant mood. For instance, Bajwa et al. (64) reported slower alternation rates in participants with major depression. However, we found no correlation between BDI or HAMA scores and alternation rate, in accord with Vierck et al. (55) that depressed mood and anxiety have little influence on BR. Thus, the presence of IEDs appears to be the predominant factor influencing BR alternation rate among IGE patients.

While this study clearly demonstrates that IEDs interfere with BR alternation and that the BR alternation rate can predict the presence of IEDs, there are several limitations. First, the IED frequency detected by a single 24-h AEEG trial may not be truly representative as IED rates can fluctuate substantially over weeks or months (65). However, in domestic hospitals and clinics, 24 h is the longest duration of EEG recording widely available. This recording interval is also widely used for the prediction of epilepsy recurrence after drug withdrawal. Dynamic monitoring of electrical activity for a week or even a month is a better choice, but it is more difficult to achieve in practical clinical research. Second, there are many types of ASMs, and their mechanisms of action are complex. Also, the ASM load affects epileptic discharges. Therefore, the effects of individual ASM treatment protocols on binocular rivalry are likely heterogeneous. This study could not evaluate the impact of different ASMs on the BR rate due to limited sample size. If possible, future studies should explore the impact of drug type and dosage on BR in a larger cohort. Third, IGE is a group of clinical syndromes, including many subtypes, with distinct etiologies. Therefore, mechanism for the slower BR rate in IGE patients may be strongly patient specificity. Again, due to the insufficient sample size, this study could not evaluate the effects of different epilepsy subtypes on BR rate. Fourth, we did not conduct extensive measures of cognitive function, such as attention and executive function. In future research, the impacts of different cognitive domains on the BR rate will be assessed.

## Conclusion

We demonstrate that an abnormally slow BR alternation rate is strongly associated with the presence of interictal discharges among IGE patients. The number of IEDs is an independent factor influencing the BR rate. These results suggest that interictal activity disrupts perceptual awareness, and that the BR may be a valuable auxiliary behavioral task to diagnose and dynamically monitor IGE patients with IEDs.

## Data Availability Statement

The original contributions presented in the study are included in the article/Supplementary Material, further inquiries can be directed to the corresponding authors.

## Ethics Statement

The studies involving human participants were reviewed and approved by Anhui Medical University Ethics Committee. Written informed consent to participate in this study was provided by the participant's legal guardian/next of kin.

## Author Contributions

JW, YJ, XY, and KW: conceived and designed the experiments. JW, WD, QW, and XY: performed the experiments. JW, WD, and YJ: analyzed the date and wrote the initial manuscript. JW and WD: finished the final manuscript with guidance from YJ and KW. All authors contributed to the article and approved the submitted version.

## Funding

This study was supported by the National Natural Science Foundation of China (81601187) and the Natural Science Foundation of Anhui Province, China (KJ2019A0289).

## Conflict of Interest

The authors declare that the research was conducted in the absence of any commercial or financial relationships that could be construed as a potential conflict of interest.

## Publisher's Note

All claims expressed in this article are solely those of the authors and do not necessarily represent those of their affiliated organizations, or those of the publisher, the editors and the reviewers. Any product that may be evaluated in this article, or claim that may be made by its manufacturer, is not guaranteed or endorsed by the publisher.
