# Supplementary material for: Interictal Activity Is Associated With Slower Binocular Rivalry in Idiopathic Generalized Epilepsy
Source: Front Neurol. 2021 Nov 10;12:720126. doi: 10.3389/fneur.2021.720126 (PMC8634877; doi:10.3389/fneur.2021.720126)
Supplement: Supplementary file 1 [file Table_1.DOCX]

Supplementary material

Table S1. The antiseizure medication of IGE patients.

| ASM | AB-AEEG (41 cases) | N-AEEG (30 cases) |
| --- | --- | --- |
| Valproic acid | 16 | 10 |
| Lamotrigine | 5 | 8 |
| Levetiracetam | 1 | 1 |
| Oxcarbazepine | 2 | 2 |
| Carbamazepine | 2 | 2 |
| Phenytoin | 1 | 0 |
| Phenobarbital | 0 | 1 |
| Valproic acid+ lamotrigine | 9 | 2 |
| Valproic acid+ levetiracetam | 0 | 0 |
| Valproic acid+ oxcarbazepine | 0 | 1 |
| Valproic acid+ carbamazepine | 2 | 0 |
| Valproic acid+ phenobarbital | 0 | 1 |
| Lamotrigine + levetiracetam | 2 | 0 |
| Lamotrigine + oxcarbazepine | 1 | 0 |
| Lamotrigine + carbamazepine | 0 | 1 |
| Phenobarbital + epilepsy ning | 0 | 1 |
